# Anesthetic considerations for cesarean delivery in a parturient with hereditary hemorrhagic telangiectasia: a case report

**DOI:** 10.1186/s40981-021-00420-4

**Published:** 2021-03-01

**Authors:** Yuji Kamimura, Toshiyuki Nakanishi, Aiji (Boku)Sato, Eisuke Kako, Motoshi Tanaka, Kazuya Sobue

**Affiliations:** 1 Department of Anesthesiology and Intensive Care Medicine, Nagoya City University Graduate School of Medical Sciences, 1 Kawasumi, Mizuho-cho, Mizuho-ku, Nagoya, 467-8601 Japan; 2 Department of Anesthesiology, Aichi Gakuin University School of Dentistry, 2-11 Suemori-dori, Chikusa-ku, Nagoya, 464-8651 Japan

**Keywords:** Hereditary hemorrhagic telangiectasia, Rendu-Osler-Weber syndrome, Pulmonary arteriovenous malformation, Cesarean delivery, Spinal magnetic resonance imaging

## Abstract

**Background:**

Hereditary hemorrhagic telangiectasia (HHT), also known as Rendu-Osler-Weber syndrome, is a rare autosomal dominant disorder characterized by mucocutaneous telangiectasia and arteriovenous malformations (AVMs). There are some anesthetic considerations for cesarean delivery in a parturient with HHT.

**Case presentation:**

A 27-year-old parturient with pulmonary hemorrhage was admitted to our tertiary perinatal center. She was first diagnosed with HHT and a cesarean delivery using spinal anesthesia at 37 weeks of gestation was initially planned. However, magnetic resonance imaging (MRI) at 32 weeks of gestation revealed spinal AVM ranging from the thoracic to the lumbar levels. Thus, elective cesarean delivery under general anesthesia was planned. The parturient had an uneventful perioperative course.

**Conclusions:**

HHT should be considered as a differential diagnosis when parturients develop pulmonary hemorrhage. In a cesarean delivery of parturients with HHT, spinal MRI during pregnancy can help in deciding the anesthetic procedure to be used.

## Background

Hereditary hemorrhagic telangiectasia (HHT), also known as Rendu-Osler-Weber syndrome, is a rare autosomal dominant disorder characterized by mucocutaneous telangiectasia and arteriovenous malformations (AVMs). The prevalence of HHT is estimated to be 1 in about 2000–40,000 [1]. In patients with HHT, pulmonary and cerebral AVMs were at least 30% and 10–20%, respectively [2], while spinal AVMs were 1–8% [3, 4].

Parturients with HHT can have a significant disease progression [2, 5], which can cause major complications [5–7]. In the report examining 262 pregnancies and 111 women with HHT, major pulmonary AVM bleeding, stroke, and maternal death were found in 1%, 1.2%, and 1% [5], respectively. Thus, parturients with HHT are at high risk and should thoroughly consider their anesthetic plan. We herein report the anesthetic management for cesarean delivery in a parturient with HHT with a ruptured pulmonary AVM and undiagnosed spinal AVMs.

## Case presentation

**Fig. 1:**
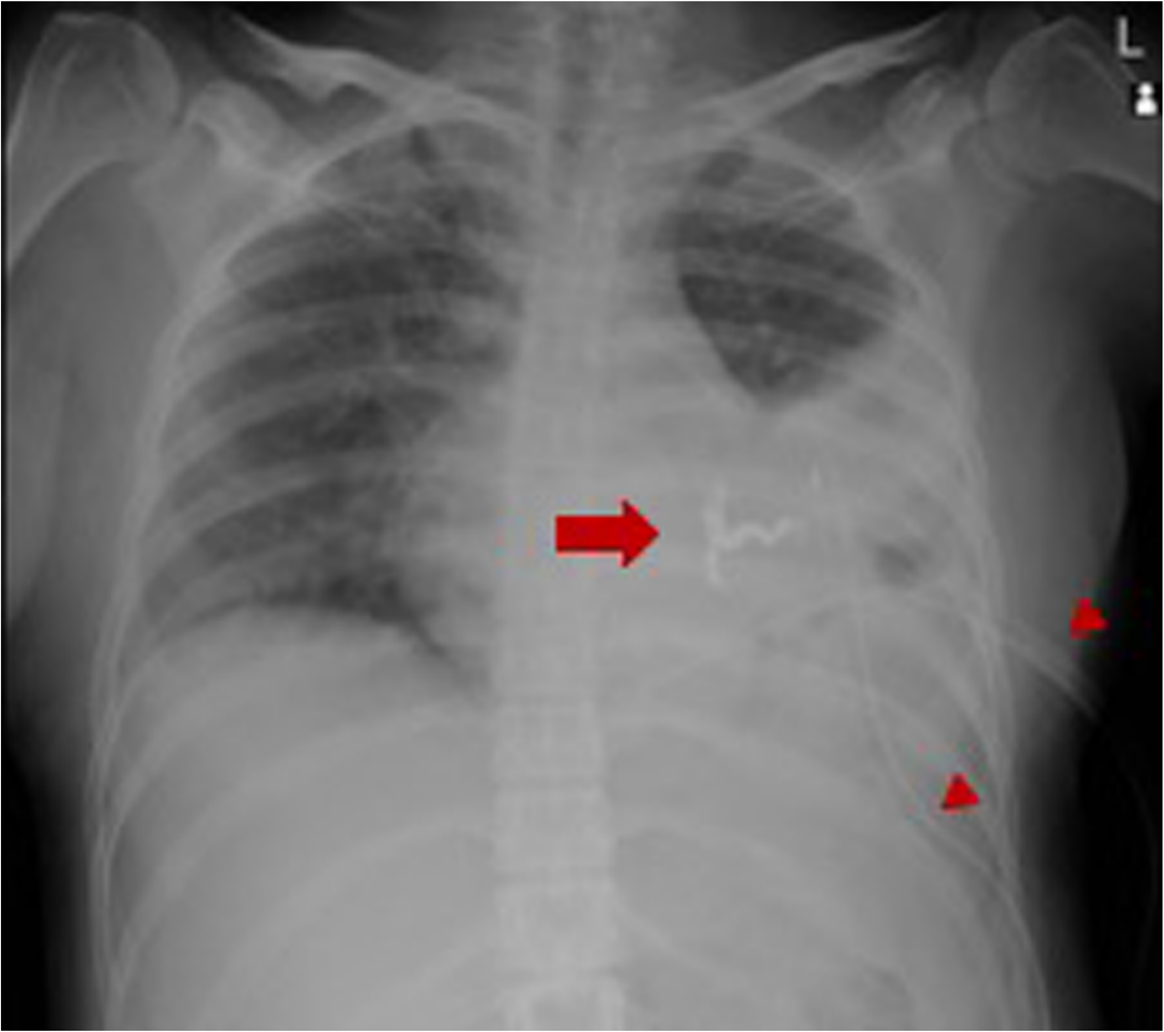
Chest X-ray imaging at 30 weeks of gestation. Embolization coils (the arrow) and chest tubes (arrowheads) are placed for the pleural hemothorax treatment due to the ruptured arteriovenous malformation

**Fig. 2:**
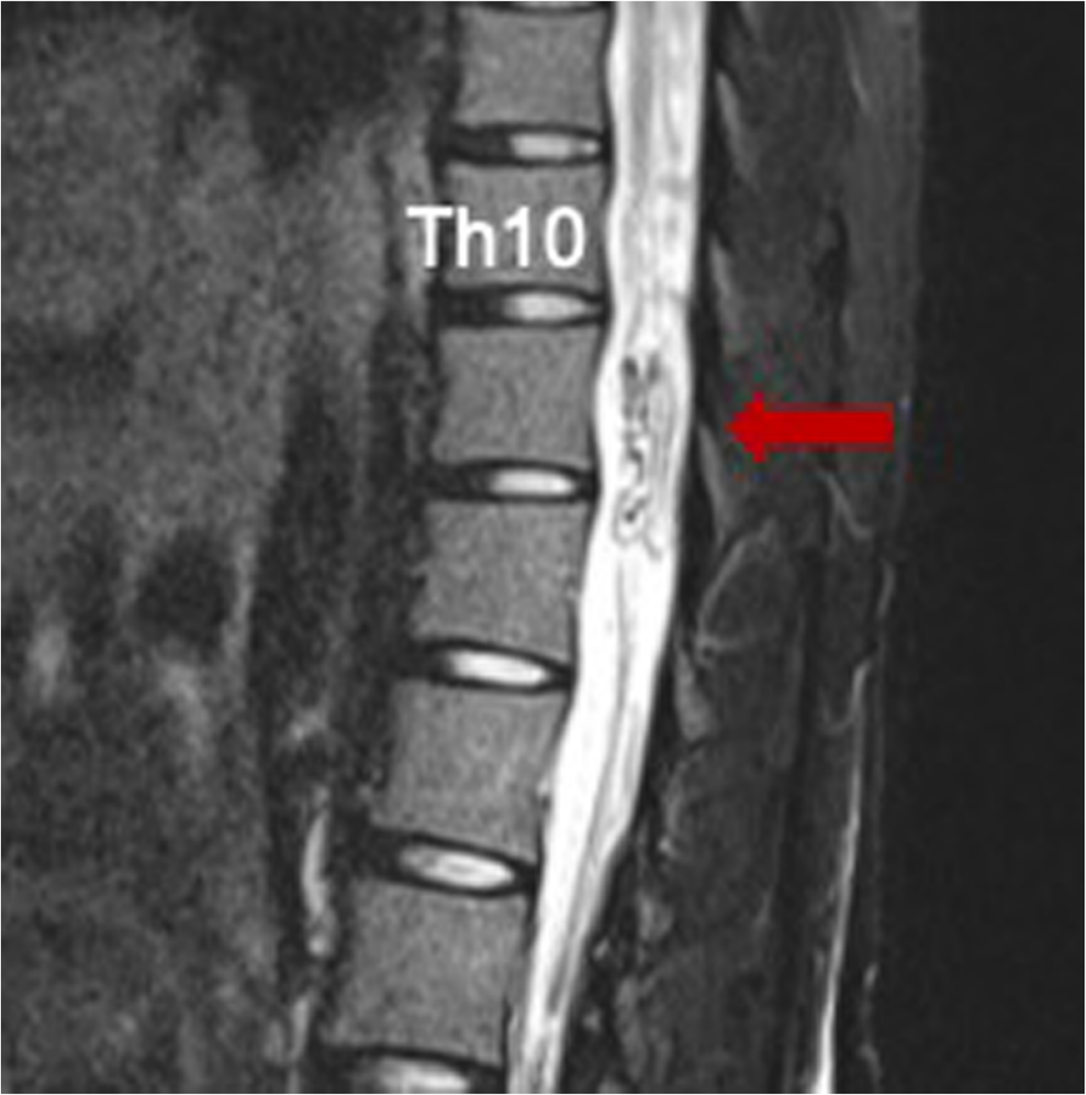
Magnetic resonance imaging (T2-weighted image) in the thoracic level at 32 weeks of gestation. Flow void (the arrow) suggests a dilated draining vein. Th10, tenth thoracic vertebra

**Fig. 3:**
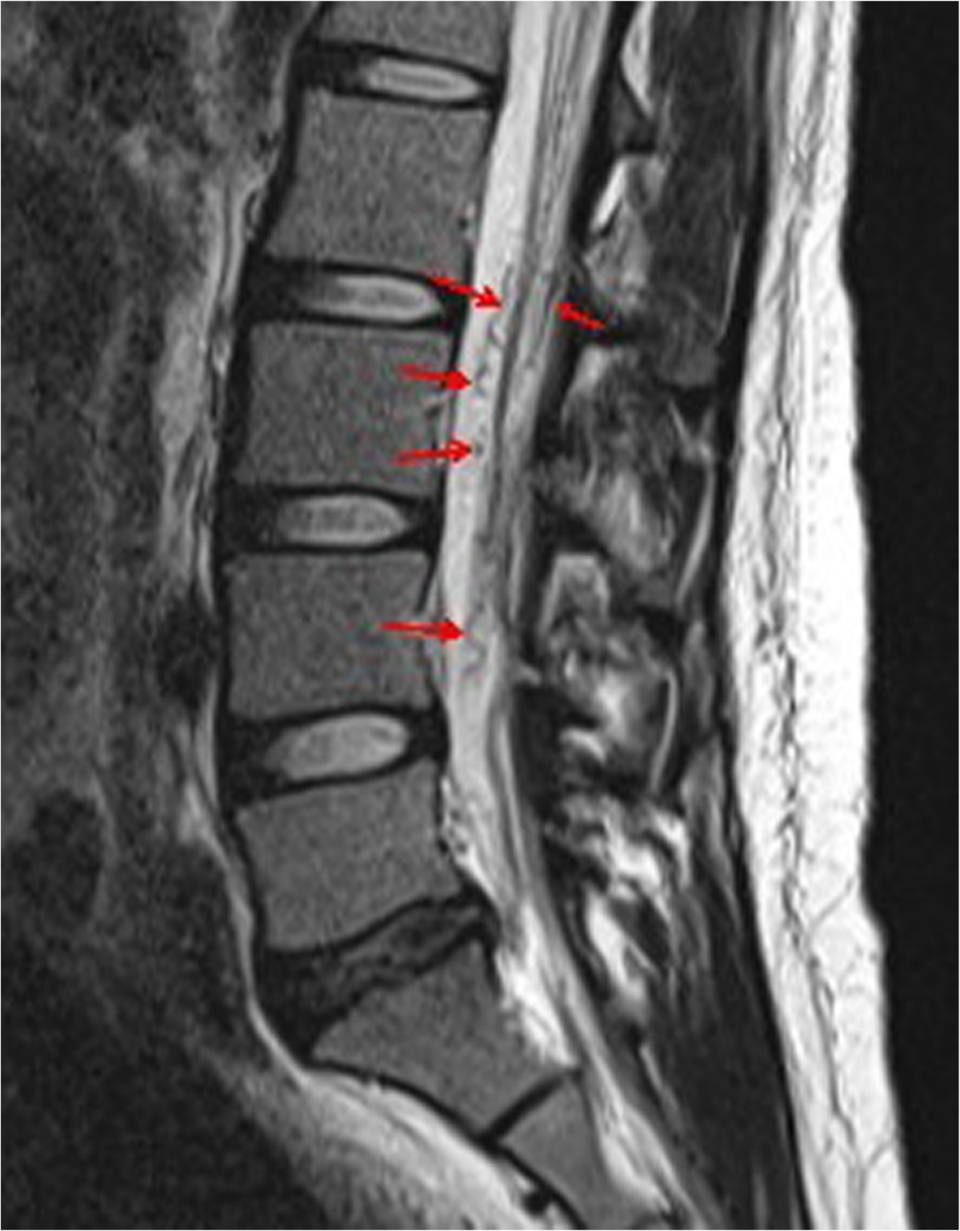
Magnetic resonance imaging (T2-weighted image) in the lumbar level at 32 weeks of gestation. Flow voids (the arrows) suggest dilated draining veins

A 27-year-old, 52-kg, 156-cm woman (G4, P1), with a previous uneventful term vaginal delivery, had epistaxis a few times a month before pregnancy. At 30 weeks of gestation, she was admitted to a local hospital’s emergency department due to excruciating back pain and dyspnea. Computed tomography revealed a ruptured pulmonary AVM (36 × 21 mm) in the left lower lobe. She was transferred to our tertiary perinatal center after embolization (Fig. 1) and was first diagnosed with HHT because of recurrent epistaxis, pulmonary AVM, and positive family history. Several discussions were held by a multidisciplinary team comprising obstetric anesthesiologists, obstetricians, pediatricians, respiratory physicians, and interventional radiologists to seek the most appropriate management. Our obstetricians preferred to avoid vaginal delivery owing to the risk of rerupture of pulmonary AVM and rupture of undiagnosed pelvic or other areas’ AVMs during vaginal delivery. Our interventional radiologists also agreed that the possibility of pulmonary AVM rerupture during vaginal delivery was not 0%, even after embolization. Therefore, elective cesarean delivery by neuraxial anesthesia at 37 weeks of gestation was planned. Preoperative examinations revealed no abnormal physical or laboratory findings. Magnetic resonance imaging (MRI) at 32 weeks of gestation revealed spinal AVM ranging from the thoracic to the lumbar levels (Figs. 2 and 3), but AVM was not present in the brain. To avoid puncturing the spinal AVM with neuraxial techniques, the anesthetic plan was changed from neuraxial anesthesia to general anesthesia. A hybrid operating where arterial embolization could be performed in case of pulmonary AVM rerupture was opted for performing the surgery.

After arriving in the operating room, general anesthesia was induced using 300 μg fentanyl, 100 mg propofol, and 60 mg rocuronium in the 30° left lateral tilt position with standard monitoring and direct left radial arterial pressure. The trachea was easily intubated by applying cricoid pressure. Mechanical ventilation settings were peak inspiratory pressure of 15 cmH2O, positive end-expiratory pressure of 5 cmH2O, frequency of 15 breaths per minute, and inspired oxygen fraction of 40%. The target range for end-tidal CO2 was set at 30–35 mmHg. Anesthesia was maintained with oxygen, air, target-controlled infusion of 2.6 μg/ml propofol, and 0.2 μg/kg/min remifentanil. She remained hemodynamically stable throughout the surgery, with SpO2 over 99%. Five minutes after the induction, a 2496-g newborn was delivered with Apgar scores of 7 and 9 at 1 min and 5 min, respectively. The umbilical arterial blood gas had pH 7.28 and base excess − 4.0 mEq/l. After the surgery, intravenous 1000 mg acetaminophen and 100 mg sugammadex were administered, and her trachea was extubated soon after confirming spontaneous breathing to avoid coughing and hypertension. The total surgical and anesthesia durations were 32 min and 54 min, respectively, with blood loss of 904 ml, including the amniotic fluid. She had an uneventful postpartum period and was discharged with her baby on the fifth postoperative day.

## Discussion

We experienced a HHT case with a ruptured pulmonary AVM and undiagnosed spinal AVMs. Since spinal MRI during pregnancy revealed AVMs ranging from the thoracic to the lumbar levels, we selected general anesthesia. This case provides two important clinical suggestions.

First, HHT should be considered as a differential diagnosis when parturients develop pulmonary hemorrhage. Although HHT is rarely diagnosed before pregnancy [5], pulmonary AVM exists in 30% patients with HHT [2], of which 1% experience significant pulmonary AVM bleeding [5]. Notably, the intrapulmonary shunt fraction increases during pregnancy [8], possibly due to increased pulmonary blood flow caused by increased blood volume and cardiac output and decreased venous distensibility due to increased progesterone levels [9, 10].

Second, spinal MRI during pregnancy can be useful in deciding the anesthesia technique in parturients with HHT. Neuraxial anesthesia was performed in 92 of 185 (50%) deliveries in women with HHT without prior screening for spinal AVMs, none of which were complicated by an epidural bleed or other serious complications [7]. On the contrary, several published cases have reported that parturients with spinal AVMs developed spinal subdural hematoma after neuraxial anesthesia [11, 12]. Changes in cord hemodynamics by spinal AVMs can cause widespread enlargement of the epidural veins, increasing the chance of trauma. Furthermore, cerebrospinal fluid loss can result in tension on the wall of spinal AVMs [13]. We believe that neuraxial anesthesia can only be administered safely in the absence of lumbar level lesions after spinal MRI.

Choosing an anesthesia technique involves balancing the risks of general and neuraxial anesthesia. The risk of general anesthesia included AVM rupture while performing anesthesia, especially that caused by a pressor response to laryngoscopy and positive pressure to mechanical ventilation. The risk of neuraxial anesthesia included puncture of spinal AVMs with neuraxial techniques. Moreover, vasodilatation produced by neuraxial anesthesia can also increase the shunt across AVMs [14]. A previous review also mentioned that spinal AVMs were contraindications to neuraxial anesthesia [6]. In our parturient, general anesthesia was performed based on the MRI findings at 32 weeks of gestation. During general anesthesia, tracheal intubation was carefully performed along with radial arterial pressure monitoring and ventilation with a small tidal volume to avoid high peak inspiratory pressure and reduce the risk of rupturing pulmonary AVMs [14].

In conclusion, we report on a case of HHT with a ruptured pulmonary AVM and undiagnosed spinal AVMs. HHT should be considered as a differential diagnosis when parturients developed pulmonary hemorrhage. In a cesarean delivery of parturients with HHT, spinal MRI during pregnancy can help in deciding the anesthetic procedure.

## Data Availability

Not applicable.

## Abbreviations

AVM: Arteriovenous malformation
HHT: Hereditary hemorrhagic telangiectasia
MRI: Magnetic resonance imaging
SpO_2_: Peripheral oxygen saturation
